# Diversity of International High-Risk Clones of *Acinetobacter baumannii* Revealed in a Russian Multidisciplinary Medical Center during 2017–2019

**DOI:** 10.3390/antibiotics10081009

**Published:** 2021-08-20

**Authors:** Andrey Shelenkov, Lyudmila Petrova, Mikhail Zamyatin, Yulia Mikhaylova, Vasiliy Akimkin

**Affiliations:** 1 Central Research Institute of Epidemiology, 111123 Moscow, Russia; mihailova@cmd.su (Y.M.); vgakimkin@yandex.ru (V.A.); 2 National Medical and Surgical Center, 105203 Moscow, Russia; lutix85@yandex.ru (L.P.); mnz1@yandex.ru (M.Z.)

**Keywords:** *Acinetobacter baumannii*, antibiotic resistance, virulence, whole-genome sequencing, international high-risk clones, genomic epidemiology

## Abstract

*Acinetobacter baumannii* is a dangerous bacterial pathogen possessing the ability to persist on various surfaces, especially in clinical settings, and to rapidly acquire the resistance to a broad spectrum of antibiotics. Thus, the epidemiological surveillance of *A. baumannii* within a particular hospital, region, and across the world is an important healthcare task that currently usually includes performing whole-genome sequencing (WGS) of representative isolates. During the past years, the dissemination of *A. baumannii* across the world was mainly driven by the strains belonging to two major groups called the global clones or international clones (ICs) of high risk (IC1 and IC2). However, currently nine ICs are already considered. Although some clones were previously thought to spread in particular regions of the world, in recent years this is usually not the case. In this study, we determined five ICs, as well as three isolates not belonging to the major ICs, in one multidisciplinary medical center within the period 2017–2019. We performed WGS using both short- and long-read sequencing technologies of nine representative clinical *A. baumannii* isolates, which allowed us to determine the antibiotic resistance and virulence genomic determinants, reveal the CRISPR/Cas systems, and obtain the plasmid structures. The phenotypic and genotypic antibiotic resistance profiles are compared, and the possible ways of isolate and resistance spreading are discussed. We believe that the data obtained will provide a better understanding of the spreading and resistance acquisition of the ICs of *A. baumannii* and further stress the necessity for continuous genomic epidemiology surveillance of this problem-causing bacterial species.

## 1. Introduction

Monitoring the spread of particular lineages of pathogenic bacteria and the associated antimicrobial resistance determinants within a particular hospital, country, or across the world represents a very important task for national and international public health institutions. Currently, such surveillance is becoming more and more dependent on next-generation sequencing (NGS) of bacterial genomes and the corresponding bioinformatics analysis pipelines [1,2,3]. Such investigations have already formed a new field called ‘genomic epidemiology’, in which methods allow to obtain huge amounts of epidemiologically and medically relevant information in a cost-, time-, and resource-efficient way [4,5,6].

During recent years, the antibiotic resistance within different species of nosocomial and community-acquired bacterial pathogens has increased to dangerous levels [7,8,9]. This problem cannot be solved without comprehensive investigations of resistance transmission mechanisms and global epidemiological surveillance.

Currently, one of the most problematic pathogens is *Acinetobacter baumannii*, accounting for about 2% of all healthcare-associated infections in USA and Europe [10] and up to 4% in Asia [11]. *A. baumannii* is a member of the ESKAPE group of bacterial pathogens, also including *Enterococcus faecium*, *Staphylococcus aureus*, *Klebsiella pneumoniae*, *Pseudomonas aeruginosa*, and *Enterobacter* spp., which are major causes of antibiotic-resistant infections worldwide [12]. It is a Gram-negative coccobacillus that is mainly responsible for causing pneumonia and wound infections associated with elevated morbidity and mortality in clinical settings [13]. The notable characteristics of *A. baumannii* include the ability to rapidly acquire multidrug-, extensive drug-, and even pandrug-resistance phenotypes [14], as well as to easily survive and transfer in the hospital environment, such as attaching to various biotic and abiotic surfaces [15]. *A. baumannii* evolution during the past five decades was mainly driven by two globally disseminated clones, GC1 and GC2 (also called IC1 and IC2, IC standing for ‘international clone’) [16]. However, six additional clonal lineages are currently generally accepted [17], and IC9 is on its way [18].

In Russia, *A. baumannii* constitutes up to 16.8% of healthcare-associated bacterial infections and exhibits a high rate of carbapenem resistance (about 77%), with the predominating clones being IC1, IC2, and IC6 [19].

Although many reports consider different clones or lineages of *A. baumannii* species to be associated with particular parts or regions of the world [13,20,21,22], the dramatically fast distribution of SARS-CoV-2 in 2020 has demonstrated that our knowledge regarding the spread of different pathogens is still limited.

Here we present the results of genomic epidemiology monitoring of *A. baumannii* in a multidisciplinary medical center in Moscow, Russia, during the period 2017–2019. Amazingly, we have revealed the isolates belonging to 5 out of 9 international clonal lineages (ICL), as well as additional isolates not clustering to any known ICL within our samples. We selected nine representative isolates for this manuscript and performed whole-genome sequencing for them using second- and third-generation (long-read) sequencing technologies. Comprehensive analyses of phenotypic and genotypic antimicrobial resistance, virulence factors, plasmids, and CRISPR arrays for the selected isolates are provided.

We believe that our data will facilitate a better understanding of *A. baumannii* spread across the world and the possible ways of acquiring antimicrobial resistance by this dangerous pathogen.

## 2. Results

### 2.1. Isolate Metadata and Typing

The metadata for the isolates and the results of their typing using the Pasteur MLST scheme, K-, and OCL-loci are presented in Table 1.

In this study, we aimed to capture the widest possible diversity of *A. baumannii* clones while keeping the number of isolates lower for the sake of presentation clarity. We selected nine isolates, six of which represented known international clones of high risk and three isolates represented singletons that could not be attributed to any ICs. Well-established ‘old’ international clones 1 and 2 [13] are presented by their general sequence types. Two isolates representing IC2 had different K- and OCL-types, so they were both included in the study to further increase the isolate diversity. 

IC4 and IC6 were also represented by their major STs [17]. It is interesting that the isolates representing two different clones, CriePir306 (IC4) and CriePir308 (IC2), were isolated from the same patient, but from different sites. However, they had the same K- and OCL-types, which is rather surprising.

CriePir307 possessed a novel sequence type that was not clustered with existing international clones by either cgMLST or MLST loci. Although it possessed the *bla*_OXA-64_ gene that is known to be the characteristic of IC7 [23], it was quite different from other IC7 isolates by cgMLST profile (see Table S1 and Figure S1). Thus, it was assigned to the most frequent Pasteur ST profile clustering in the same clonal complex (CC152). CriePir298, which belonged to IC7, possessed a general MLST sequence type for this clone—ST25 [17]. However, it had a rather rare capsular type—KL116 [24].

CriePir254 and CriePir309 also did not belong to the known international clonal lineages, but could be attributed to CC252 and CC132, respectively.

**Figure 1:**
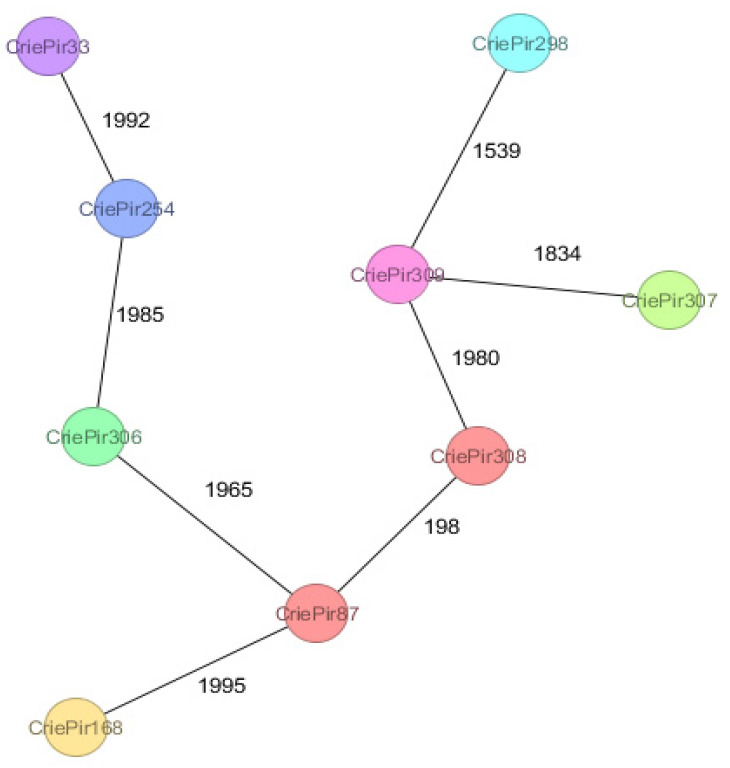
The minimum spanning tree for the isolates studied based on cgMLST profiles. The numbers indicate the amount of different alleles between the pairs of corresponding isolates. Close isolates (CriePi87 and CriePi308) are shown in the same color; other isolates are not close to each other.

The minimum spanning tree for the isolates is presented in Figure 1. It is not surprising that the isolates are located very far from each other in terms of their cgMLST profiles since the aim of this study was to capture the maximal possible diversity. Only the isolates CriePir87 and CriePir308 belonging to IC2 were comparatively close to each other (198 allele differences). 

We also built a cgMLST tree for our isolates and the whole set of RefSeq isolates (accessed on 20 March 2021), trying to infer the possible spreading information. A subset of this tree, including the closest matches from RefSeq to our isolates in terms of the number of cgMLST allele differences, is provided in the Figure S1.

As we can see from this tree, the isolates with unusual profiles, such as CriePir254 (rare ST, no IC) and CriePir307 (novel ST, no IC), do not have close relatives in terms of the number of allele differences. Moderately close neighbors were revealed for CriePir168 (IC1, GCA_000830055.1, Australia, distance = 187), CriePir308 (IC2, GCA_000314655.1, USA, distance = 50), CriePir306 (IC4, GCF_003583665.1, Spain, distance = 147), and CriePir33 (IC6, GCF_003948375.1, USA, distance = 87). However, this information does not allow inferring the spreading routes of the isolates, and more genomic data is required to achieve this goal.

Complete cgMLST profiles for all our isolates and their closest matches from RefSeq are presented in Table S1.

### 2.2. Antimicrobial Resistance

**Figure 2:**
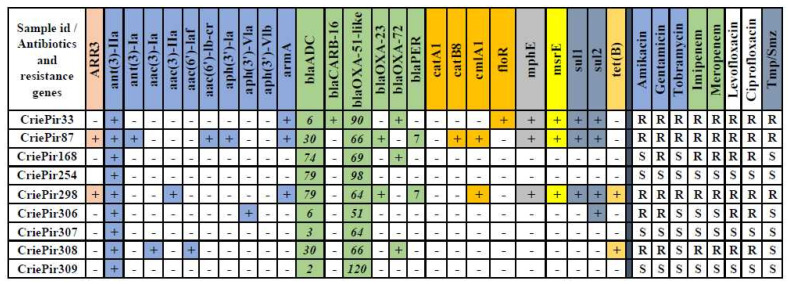
Antimicrobial resistance of the *A. baumannii* isolates studied. Corresponding antibiotics and resistance genes are filled with the same colors. The numbers for the *bla* genes indicate the corresponding variants for the sake of brevity. Tmp/Smz—trimethoprim/sulfamethoxazole.

Phenotypic characterization and the genotypic resistance determinants of the isolates studied are presented in Figure 2.

As we can see from Figure 2, the isolates form three groups containing three members each: multidrug-resistant (CriePir33, CriePir87, and CriePir298), which exhibit resistance to all antibiotics from the panel; non-resistant (CriePir254, CriePir307, and CriePir309, possessing only intrinsic oxacillinase genes); and intermediate (CriePir168, CriePir306, and CriePir308), having resistance only to some of the antibiotics tested. Five isolates were carbapenem-resistant, and the likely mechanism of such a resistance is expression of *bla_OXA-_*_23_ (CriePir87 and CriePir298) and *bla_OXA-72_* (CriePir33, CriePir168, and CriePir308) carbapenemase genes. In addition, the *bla_OXA-_*_23_-carrying isolates also encoded PER-7 extended spectrum *β*-lactamase (ESBL), which was reported to demonstrate high activity against broad-spectrum cephalosporins in *A. baumannii* [25].

The resistant isolates also included genes and gene clusters providing resistance to aminoglycosides (for example, *armA*), sulfamethoxazole (*sul1* and *sul2*), chloramphenicol (*cmlA1*), and tetracycline (*tet(B)*). However, the latter two antimicrobials were not included in the panel.

Interestingly, CriePir33 possessed the *bla_CARB_*_-16_ gene representing a rather rarely occurring *bla_CARB_*_-5_-like class A beta-lactamase gene, which was first revealed in *Acinetobacter pittii* [26]. The enzyme encoded by *bla_CARB_*_-16_ differs only by one amino acid substitution from *bla_CARB_*_-5_.

In general, the isolates demonstrated very good compliance between the phenotypic and genotypic characteristics of their antimicrobial resistance. The group of susceptible isolates included only intrinsic blaADC and blaOXA-51-like oxacillinases, as well as the *ant(3)-IIa* gene, while the isolates that exhibited the multidrug-resistant phenotype possessed the largest number of acquired resistance genes (8 for CriePir33, 13 for CriePir87, and 11 for CriePir298, respectively).

### 2.3. Virulence Genes

**Figure 3:**
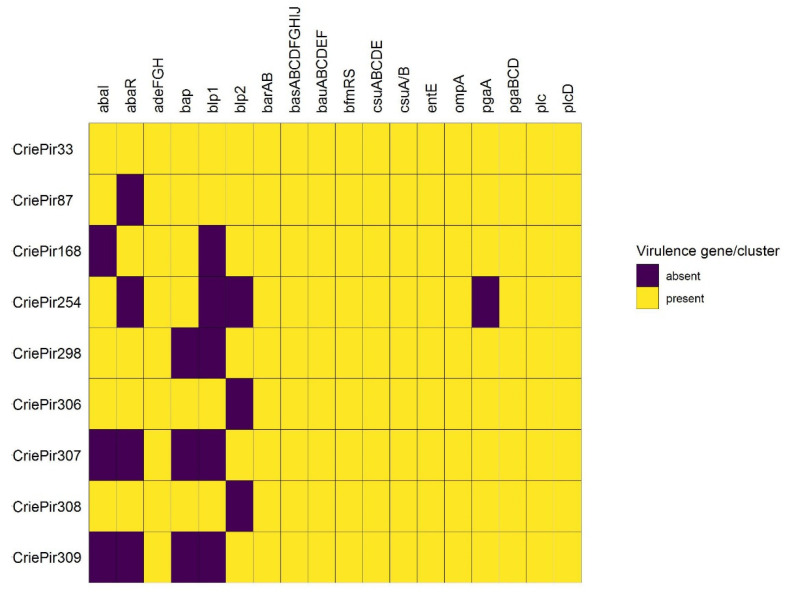
Virulence factors of the *A. baumannii* isolates studied. Genes constituting the same cluster presented in all isolates were combined for the sake of brevity. abaI, abaR—components of the quorum sensing system; adeFGH—efflux pump; bap—biofilm-associated protein; blp1,blp2—bap-like proteins; barAB—siderophore efflux system of the ABC superfamily; basABCDFGHIJ—proteins involved in biosynthesis of acinetobactin; bauABCDEF—receptor for ferric-acinetobactin complexes; bfmRS—two-component signal transduction system; csuA/BABCDE—Csu pili; entE—enterobactin biosynthesis; ompA—outer membrane protein; pgaABCD—biofilm formation locus; plc, plcD—phospholipase genes.

The distribution of the virulence genes in the isolates studied is shown in Figure 3.

The virulence gene sets of all isolates were quite similar. They mainly included the factors involved in biofilm formation (*adeFGH*, *csu*, and *pga* clusters) as well as the *bau* and *bas* clusters involved in the iron acquisition system and acinetobactin functioning.

CriePir306 and CriePir308 included all 39 factors revealed, while the other isolates lacked from one to four virulence genes each. Interestingly, we have not revealed any virulence plasmids, and all virulence genes were located on chromosomes.

The list of NCBI accession numbers for the virulence genes revealed is shown in Table S2.

### 2.4. Plasmids

The isolates had from one to six plasmids each (Table S3). However, these plasmids usually did not carry the resistance genes, except for CriePir298, which possessed the plasmid containing all resistance determinants, excluding *bla_OXA-23_* and the intrinsic genes, as well as CriePir33, CriePir168, and CriePir308, the latter two of which carried two copies of *bla_OXA-72_* of plasmid origin. Interestingly, the plasmids of CriePir168 (length = 10,878 bp) and CriePir308 (length = 10,879 bp) had essentially the same sequences except for several deletions that might result from long-read assembly algorithm imperfections. These two isolates belonged to different clonal lineages (ST1 and ST2, respectively) and were isolated with an interval of 2 years. However, both of them were found in the same clinical department (ICU), and thus the persistence of the *bla_OXA-72_*-carrying plasmid within this unit could be suggested. Another interesting fact was that these plasmids had 99.7% identity with the pAB120 plasmid (Genbank accession CP031446.1) of the MDR-UNC *A. baumannii* isolate (ST2), which caused a fatal case of necrotizing fasciitis in a USA hospital in 2019 [27]. 

CriePir298 included three plasmids, the smallest of which (length = 2343) had exactly the same sequence as pA85-1a (Genbank accession CP021784.1) from the A85 *A. baumannii* strain isolated in Australia in 2003. This plasmid was also found in several other isolates of IC1 [28], but its functional properties were not described. However, CriePir298 belonged to IC7. A brief description of the plasmids is provided in Table 2.

### 2.5. CRISPR Arrays and CRISPR/Cas Systems

CRISPR arrays were revealed in seven out of nine isolates (except CriePir87 and CriePir308). However, at least five repeats with an evidence level = 4 were found only in four isolates (CriePir168, CriePir298, CriePir307, and CriePir309), and all of them except the latter possessed a full CAS-Type IF system. Interestingly, these CAS systems had different subtypes, namely, IF-1 for CriePir168 and CriePir298, and IF-2 for CriePir307. Detailed analysis of the differences between these two subtypes lies beyond the scope of this manuscript.

Interestingly, although CriePir309 had a highly confident CRISPR array with 17 repeats, it was also the only isolate carrying an anti-CRISPR element, which could prevent the development of a functional CRISPR/Cas system.

Most repeats and all *cas* genes were located on the chromosomes. CriePir168 had one array on the short plasmid (length = 2372), and CriePir308 had one of seven arrays on another plasmid (length = 14,128). However, both of these repeats had a low evidence level.

Detailed characteristics of the CRISPR elements, including the chromosome positions, repeat sequences, and accession numbers for the *cas* genes revealed, are provided in Table S4.

## 3. Discussion

In this manuscript, we presented the genomic epidemiology investigation of a diverse *A. baumannii* population within a multidisciplinary medical center in Moscow, Russia, for the period 2017–2019. We carefully selected the isolates representing the unusual international clone variability, as well as additional isolates belonging to singleton clones, and performed their long-read sequencing in order to obtain highly accurate chromosome sequences and delineate plasmids. Hybrid short- and long-read assemblies allowed us to improve the prediction of virulence and the resistance genomic determinants, as well as to retrieve additional information required for application of genomic epidemiology tools such as cgMLST analysis.

In order to perform the epidemiological surveillance and track the spreading of pathogenic bacteria within a particular healthcare facility, some geographical region, or across the world, a reliable typing scheme based on molecular or genomic characteristics of the isolates is required. Many typing schemes are already provided for pathogenic bacteria, including *A. baumannii*. Exemplary schemes/profiles comprise the ones based on the nucleotide frequency matrices for genomic sequences [29], CRISPR sequences [30], multilocus sequence typing (MLST) [31,32] based on seven housekeeping genes, MLST/KL loci [33], and core genome MLST (cgMLST) based on 2390 genes [34]. The latter was recently successfully applied for an outbreak investigation [35]. In addition, intrinsic carbapenemase *bla_OXA-51_*-like gene variants were also proposed as a tool for *A. baumannii* identification and typing [36].

However, one of the most commonly used concepts for molecular epidemiology investigations is epidemic clonal lineages, or simply ‘international clones’, which represent genetically distinct populations of *A. baumannii* successfully spreading in different geographic locations [37]. Eight international clones were defined earlier [17], and the ninth was introduced recently [18]. The first three clones (IC1–IC3) are distributed worldwide, with IC1 and IC2 also known as global clones (GC), while the rest were sometimes defined as regional, or even endemic clones [13,20,21,22].

In contrast to this, we revealed an isolate belonging to IC7 in our hospital, although this clone was recently reported to be prevalent in South America and not in Europe [22]. Although our initial set of isolates contained less than 50 sequenced samples, we managed to reveal five different ICs and three singleton isolates not attributed to known clonal lineages. One of these isolates belonged to IC7: CriePir298 possessed the founder sequence type ST25, the members of which were revealed in Bolivia [22]. It is interesting that the ST1487 possessed by CriePir307 is rather different from ST25 in its allelic profile, and they do not cluster according to the eBURST analysis; at the same time, these two isolates share the same intrinsic OXA-51 variant, namely, OXA-64, and thus likely belong to the same lineage IC7 [23]. However, a large number of MLST and cgMLST allele differences did not allow to assign CriePir307 to IC7, and it was closer to the members of CC152. Another intriguing fact is that the resistance characteristics of CriePir298 and CriePir307 are completely different—the former was resistant to all antibiotics from the panel, while the latter was susceptible to all antibiotics tested. However, genomic analysis revealed that most resistance genes of CriePir298 were located on the resistance plasmid, which was very similar to pMC1.1 (Genbank accession MK531536.1) revealed in Bolivia in the investigation mentioned above [22].

Historic global clones were represented in our set by IC1 (CriePir168) and IC2 (CriePir87 and CriePir308). IC4, possessing the *bla_OXA-_*_51_ intrinsic gene, was revealed in CriePir306 (ST15). This clone was previously found in South America [38], but it is also present in Europe [39]. IC6, which was once considered to be a Russian endemic [20] and constituted about 25% of clinical isolates in Russia in 2015–2016 [19], was presented by CriePir33, a carbapenem-resistant isolate carrying, among others, *bla_CARB_*_-16_ and *bla_OXA-72_* resistance determinants. This complies with the observation that IC6 isolates from Russia usually obtain carbapenem resistance by acquiring class D carbapenemase genes and not by other mechanisms such as intrinsic gene mutations [19]. Unfortunately, the cgMLST comparison with the reference isolates did not reveal any clues regarding the routes of their spreading across the world due to the ubiquitous presence of widespread ICs in various geographical regions.

Finally, two isolates, CriePir254 and CriePir309, could not be attributed to known international clones. CriePir309, carrying *bla_OXA-120_* intrinsic beta-lactamase, can be assigned to the known complex CC132, although it is also rather close to CC33 [40], while CriePir254 is close to CC252. CriePir309 has a very rare ST911; currently, no full genomic sequences for the isolates of this sequence type are available in Genbank, and there is no information, except a definition, for this ST in the PubMLST database (https://pubmlst.org, accessed in 23 April 2021). This isolate was obtained from the only patient involved in the study living outside the Moscow region, and it possessed various interesting properties. For example, it did not carry resistance genes except intrinsic variants of the *bla_ADC_* and *bla_OXA-51_*-like genes, and it was the only isolate having anti-CRISPR proteins encoded (AcrIF11). At the same time, it carried several virulence gene clusters (*adeFGH*, *basABCDFGHIJ*, *csuABCD,* but not *bap*), which made it more similar to the strains causing community-acquired infections. CriePir254 and CriePir307 were similar to CriePir309 in this sense, since they also did not have resistance determinants. Thus, we can conclude that the isolates from our dataset that appeared to be rather distant from the known international high-risk clones were in fact less dangerous in terms of their antibiotic resistance.

In summary, our results confirm that in the era of globalization and rapid pathogen spread across the world, the concept of endemic clones becomes obsolete. For example, we revealed IC7 in Russia, and IC6, which was previously attributed to Europe, was recently found in Brazil [41]. In addition, a recent publication of the spatio-temporal distribution of *A. baumannii* in Germany showed that the isolates belonging to IC1, 2, 4, 6, and 7 were revealed during 2000–2018 [39], which corresponds to our dataset. However, the remarkable feature of our study is the discovery of such an IC variety within one hospital during a limited period of time, and for the patients living in the Moscow region and without a history of international travelling shortly before their hospital admission. This finding will allow developing updated prevention strategies and epidemiological measures to limit further high-risk clone spreading.

While epidemiological data is vitally important for studying the spread of any pathogenic bacteria, the information regarding the presence of antibiotic resistance and virulence factors in the isolates studied, as well as the possible mechanisms of their transfer, is no less important for developing the prevention measures. Sequencing on MinION and hybrid short-long read assembly allowed us to identify the locations of genes encoding oxacillinases and other resistance genes. The *bla_OXA-_*_23_ genes of two isolates (CriePir87 and CriePir298) were located on the chromosome, while *bla_OXA-72_* for each of the three isolates (CriePir33, CriePir168, and CriePir308) were revealed on plasmids. Such a distribution complies well with previous studies [36,42].

In general, the antibiotic resistance genes of our isolates were not located on plasmids, except for CriePir298 and the three isolates mentioned in the previous paragraph. However, plasmid investigation can contribute not only to antimicrobial resistance studies but also to tracing the spread of the pathogens across the world, although the plasmid complement is not a reliable measure of relatedness [28]. For example, in our isolates we revealed the plasmids identical to the ones of clinical isolates from the USA and Australia. However, these isolates belonged to different clonal lineages than ours, so the possible ways of plasmid transferring between them cannot be easily reconstructed. 

In contrast, we have not revealed any virulence plasmids in our isolates, and all the virulence genes were located on the chromosomes.

Virulence factors were represented in all of the isolates by the members of *csu* and *pga* clusters involved in biofilm formation [43,44], as well as the members of *bau* and *bas* clusters taking part in the iron acquisition system and acinetobactin transport and biosynthesis, respectively [45,46]. In addition, all isolates included the genes of the *adeFGH* efflux pump, the overexpression of which was also found to be associated with biofilm formation [47]. These genes are rather common for clinical *A. baumannii* isolates and, together with the other genes revealed (e.g., *bap* for CriePir87 and CriePir306), provide the environmental persistence for them [14]. In addition, all isolates contained the *ompA* gene, encoding a major component of outer membrane vesicles, which was considered to be a crucial virulence factor of *A. baumannii* [48]. Interestingly, the *bap* gene was revealed only in the isolates lacking the CRISPR/Cas system; this could be the result of preventing horizontal gene transfer by this system. 

The sets of virulence factors were similar for all isolates, except for *abaR*, which was not revealed in the fully susceptible isolates (CriePir254, CriePir307, and CriePir309) and CriePir87, and *abaI*, which was not found in CriePir168, CriePir307, and CriePir309. These genes are involved in quorum sensing and may contribute to motility and host–pathogen interaction [45]. The presence of *abaI*/*abaR* was positively correlated with bacterial resistance rates [49], and thus their absence in susceptible isolates complies with this finding. However, additional investigations are needed to elucidate the possible mechanisms since such a correlation was not perfect for our isolates.

Finally, we can conclude that obtaining extensive data on the spreading of particular strains, high-risk clones, antimicrobial resistance, and virulence factors across a particular hospital, country, and region greatly facilitates developing the epidemiological measures for preventing an exponential increase in MDR *A. baumannii* strains, as well as other pathogenic bacterial species. Unfortunately, these measures are not sufficient for fighting the resistant bacteria in clinical settings. Possible holistic approaches to cope with this problem could include antibiotic stewardship [50], developing novel antibiotics [51], using bacteriophages, and other antibacterial moieties, such as antibodies, synthetic membrane-active agents, or antimicrobial peptides [52,53,54,55].

## 4. Materials and Methods

### 4.1. Determination of Antibiotic Susceptibility

Species identification for all isolates was performed by time-of-flight mass spectrometry (MALDI-TOF MS) using the VITEK MS system (bioMerieux, Marcy-l’Étoile, France), and the susceptibility to antimicrobials was determined by the disc diffusion method using the Mueller–Hinton medium (bioMerieux, Marcy-l’Étoile, France) and disks with antibiotics (BioRad, Marnes-la-Coquette, France), and by the Minimum Inhibitory Concentration (MIC) method on a VITEK 2 Compact 30 analyzer (bioMerieux, Marcy-l’Étoile, France). The antibiotics panel included the following drugs: amikacin, gentamicin, tobramycin, imipenem, meropenem, levofloxacin, ciprofloxacin, and trimethoprim/sulfomethoxazole. These antimicrobial compounds reflected those agents used for human therapy in the Russian Federation. We used the EUCAST clinical breakpoints, version 11.0 (https://www.eucast.org/clinical_breakpoints/, accessed on 20 December 2020), to interpret the results obtained.

### 4.2. DNA Isolation, Sequencing, and Genome Assembly

Nine samples were obtained from eight patients (5 males and 3 females) in various sources and hospital departments (Table 1) of a multidisciplinary federal medical center in Moscow, Russia, during the period 2017–2019. The age of the patients involved in this study ranged from 27 to 62 years with a median equal to 56.

The total number of isolates involved in the initial screening was 145, and 49 of them were sequenced. Earlier we have investigated the properties of the CRISPR/Cas arrays and systems for some of these isolates and a set of reference isolates from RefSeq [56]. Then we carefully selected nine isolates from the initial set, which represented the diversity of the *A. baumannii* international clones revealed in the hospital, and performed long-read sequencing for them. Our aim was not to capture the diversity of all strains found in the hospital during the study period, but rather to investigate the spread of international high-risk clones and to check the hypothesis of their endemicity for a particular region or country. Long-read sequencing allowed us to obtain the precise genome and plasmid structures, as well as to verify the locations of antibiotic resistance and virulence determinants, and to obtain complete cgMLST profiles for the selected representative isolates.

Genomic DNA was isolated with the DNeasy Blood and Tissue kit (Qiagen, Hilden, Germany). A Nextera™ DNA Sample Prep Kit (Illumina^®^, San Diego, CA, USA) was used for paired-end library preparation, and whole-genome sequencing (WGS) of the isolates on Illumina^®^ Miseq and Hiseq platforms (Illumina^®^, San Diego, CA, USA).

Additional WGS was performed using the Oxford Nanopore MinION sequencing system (Oxford Nanopore Technologies, Oxford, UK). The same genomic DNA was used to prepare the MinION library with the Rapid Barcoding Sequencing kit SQK-RBK004 (Oxford Nanopore Technologies, Oxford, UK). The amount of initial DNA used for barcoding kit was 400 ng for each sample. The libraries were prepared according to the manufacturer’s protocols, and were sequenced on R9 SpotON flow cell by initiating the standard 24 h sequencing protocol using the MinKNOW software (Oxford Nanopore Technologies, Oxford, UK).

Base calling of the raw MinION data was performed using Guppy Basecalling Software version 4.4.1 (Oxford Nanopore Technologies, Oxford, UK), and demultiplexing was made using Guppy barcoding software version 4.4.1 (Oxford Nanopore Technologies, Oxford, UK). Hybrid assemblies were obtained using short- and long-reads by Unicycler version 0.4.9-beta [57].

Genome assemblies were uploaded to NCBI Genbank under the following accession numbers: JAEPWJ000000000 (CriePir33), JAEPWI000000000 (CriePir87), JAEPWG000000000 (CriePir168), JAHHIS000000000 (CriePir254), JAEPWB000000000 (CriePir298), JAEPVY000000000 (CriePir306), JAEPVX000000000 (CriePir307), JAEPVW000000000 (CriePir308), and JAEPVV000000000 (CriePir309).

### 4.3. Data Processing

The genomes assembled were processed using a custom software pipeline described earlier [33]. We used the Resfinder 4.0 database for antimicrobial gene identification (https://cge.cbs.dtu.dk/services/ResFinder/, accessed on 20 April 2021). VFDB [58] was used to search for the virulence factors http://www.mgc.ac.cn/VFs/main.htm (accessed on 20 April 2021). 

Isolate typing was first performed by MLST using the Pasteur scheme. It was chosen for typing since, according to the Oxford scheme, five isolates possessed undetectable sequence types (STs) with a duplicated *gdhB* locus. Additional classification was made by using capsule synthesis loci (K-loci) [59] and lipooligosaccharide outer core loci (OCL) [60]. Detection of cgMLST profiles was performed using MentaList software (https://github.com/WGS-TB/MentaLiST, version 0.2.4, accessed on 10 June 2021), and the minimum spanning tree was build using PHYLOViz online (http://online.phyloviz.net, accessed on 10 June 2021).

CRISPRCasFinder [61] was used to identify the presence of CRISPR/Cas systems and spacers in the genomes studied. Anti-CRISPR elements were searched in AcrBank http://cefg.uestc.cn/anti-CRISPRdb (accessed on 20 April 2021).

## 5. Conclusions

In this study, we performed third-generation sequencing-based genomic epidemiology surveillance of clinical *A. baumannii* isolates from a multidisciplinary medical center in Moscow, Russia, obtained during 2017–2019. Surprisingly, we revealed that our isolates included 5 of the 9 commonly defined international clones of this important nosocomial pathogen. In addition, three isolates possessed singleton sequence types not clustered with the known lineages, including ST911, for which whole-genome data are not available yet. We presented a detailed analysis of the phenotypic antimicrobial resistance and genomic resistance determinants for all the isolates studies, as well as additional data for virulence factors, plasmids, and CRISPR arrays. We believe that these data will facilitate a better understanding of the clonal spreading and resistance acquisition of *A. baumannii* and further highlight the necessity of continuous genomic epidemiology surveillance for this problematic pathogen.

## Figures and Tables

**Table 1:** The origin and typing of the clinical *A. baumannii* isolates studied.

Sample id	Patient Code	Isolation Date	Clinical Department	Locus	MLST	OCL-Type	KL-Type	IC
CriePir33	P1	03.05.2017	Traumatology	Wound	ST78	OCL1	KL3	IC6 (CC78)
CriePir87	P2	04.07.2017	Surgery	Soft tissue abscess	ST2	OCL1	KL33	IC2 (CC2)
CriePir168	P3	03.12.2017	ICU	Urine	ST1	OCL1	KL17	IC1 (CC1)
CriePir254	P4	24.08.2018	Surgery	Bile	ST370	OCL1	KL25	CC252
CriePir298	P5	10.09.2019	CNS Rehabilitation	Urine	ST25	OCL6	KL116	IC7 (CC25)
CriePir306	P6	05.08.2019	ICU	BAL	ST15	OCL7	KL9	IC4 (CC15)
CriePir307	P7	29.06.2019	ICU	BAL	ST1487 *	OCL2	KL45	CC152
CriePir308	P6	22.08.2019	ICU	CVC	ST2	OCL7	KL9	IC2 (CC2)
CriePir309	P8	31.08.2019	CNS Rehabilitation	Urine	ST911	OCL6	KL14	CC132

ICU—intensive care unit; CVC—central venous catheter; BAL—bronchoalveolar lavage; *—novel ST identified by us.

**Table 2:** Plasmid characteristics of the isolates studied.

Sample Id	Number of Plasmids	Plasmid Sizes	Virulence/Resistance Determinants in Plasmids	Related Plasmids from Other Isolates
CriePir33	1	17,765	*bla_OXA-72_*	pIBAC_oxa58_20C15, KY202458.1
CriePir87	1	11,194	-	pUnnamed1, CP035673.1
CriePir168	3	1217–10,878	*bla_OXA-72_*	pAB120, CP031446.1
CriePir254	2	10,427–90,326	-	pGFJ6, CP016902.1
CriePir298	3	2343–183,139	*mph(E), msr(E), armA, sul1, sul2, blaPER-7, ARR-3, aph(6)-Id, aph(3′′)-Ib, aac(3)-IIa, tet(B)*	pA85-1a, CP021784.1
CriePir306	4	2845–80,829	-	pA1296_1, CP018333.1
CriePir307	6	2278–114,430	-	pABAY15001_6E, MK386684.1
CriePir308	1	10,879	*bla_OXA-72_*	pAB120, CP031446.1
CriePir309	2	11,195–94,551	-	pTS134338, CP042210.1

## Data Availability

The bacterial genomes presented in this study are openly available in NCBI Genbank under the following accession numbers: JAEPWJ000000000 (CriePir33), JAEPWI000000000 (CriePir87), JAEPWG000000000 (CriePir168), JAHHIS000000000 (CriePir254), JAEPWB000000000 (CriePir298), JAEPVY000000000 (CriePir306), JAEPVX000000000 (CriePir307), JAEPVW000000000 (CriePir308), and JAEPVV000000000 (CriePir309).

## Supplementary Materials

The following are available online at https://www.mdpi.com/article/10.3390/antibiotics10081009/s1, Table S1. cgMLST profiles for clinical *A. baumannii* isolates included in the study, Table S2. Accession numbers for the virulence genes revealed in the isolates studied, Table S3. Plasmid description for the isolates studied, Table S4. Description and statistics for the CRISPR arrays revealed in clinical *A. baumannii* isolates included in the study, Figure S1. cgMLST tree for the isolates under study and their closest matches from RefSeq database.
