# Supplementary figures and images for: Diversity of International High-Risk Clones of Acinetobacter baumannii Revealed in a Russian Multidisciplinary Medical Center during 2017–2019
Source: Antibiotics (Basel). 2021 Aug 20;10(8):1009. doi: 10.3390/antibiotics10081009 (PMC8389025; doi:10.3390/antibiotics10081009)

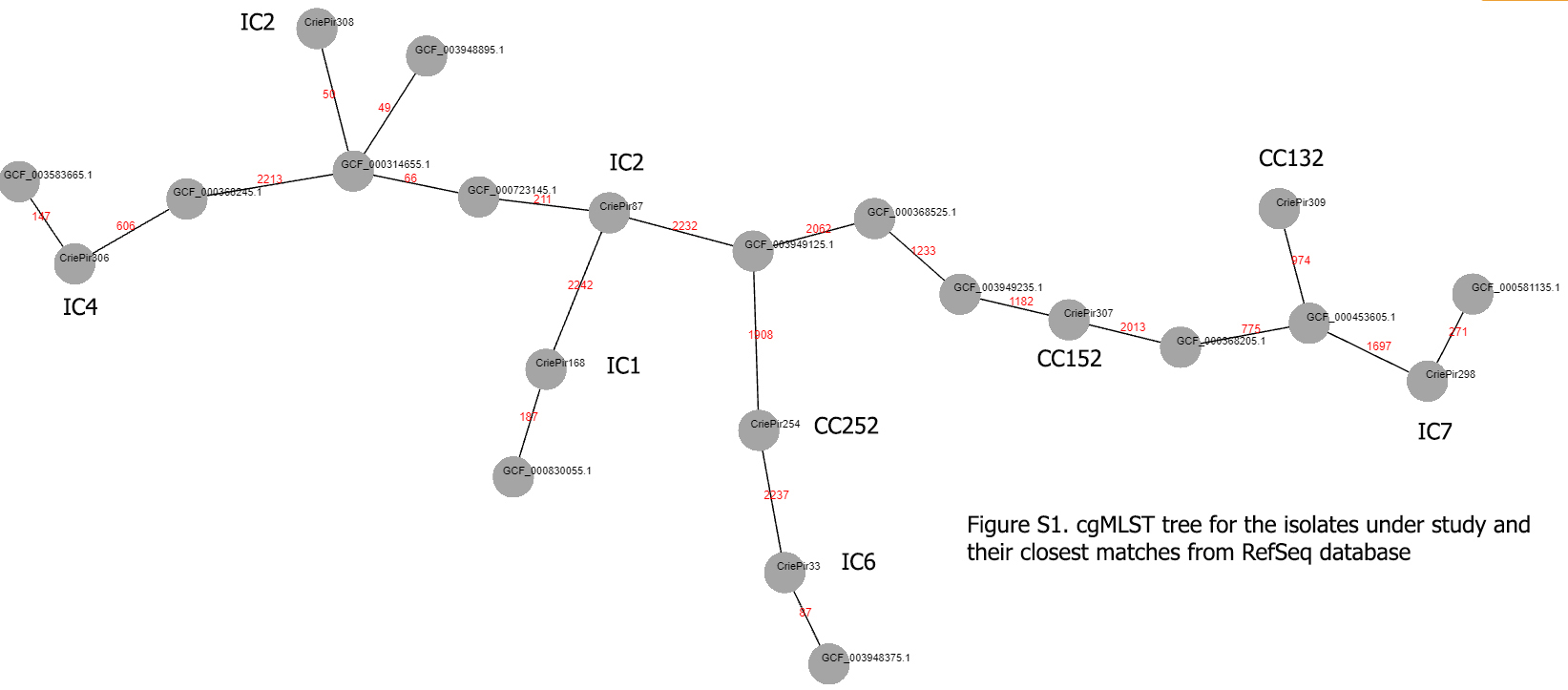

Supplement: Supplementary file 1 [file antibiotics-10-01009-s001.zip › figS1.jpg]
